# The impact of project-based learning on university physics education: enhancing cognitive skills and core competencies

**DOI:** 10.3389/fpsyg.2025.1495105

**Published:** 2025-09-15

**Authors:** Chenglong Wang, Lirong Song, Jun Jiang

**Affiliations:** School of Educational Sciences, Harbin Normal University, Harbin, China

**Keywords:** project-based learning, university physics, conceptual understanding, problem-solving, student engagement, collaborative learning, practical skills, higher education

## Abstract

**Background:**

Project-Based Learning (PBL) has been proposed as an alternative to traditional lecture-based instruction in university physics courses. This study examines the effects of PBL on undergraduates' conceptual understanding, problem-solving skills, practical competencies, engagement, and motivation.

**Methods:**

A mixed-methods quasi-experimental design was employed with 150 physics majors from three universities in Harbin, China. Participants were stratified by key demographics and baseline physics knowledge, then randomly assigned to a PBL group (*n* = 75) or a lecture-based control group (*n* = 75). Quantitative data were gathered via standardized pre- and post-tests; effect sizes were calculated (Cohen's d). Qualitative data were obtained through structured classroom observations and semi-structured interviews. Multiple regression analyses—controlling for baseline knowledge—evaluated predictors of post-test performance (R^2^, significance tests), with diagnostic checks for normality and multicollinearity (VIF).

**Results:**

The PBL group demonstrated significantly greater gains in conceptual understanding (Cohen's d = 1.85) and problem-solving ability than the control group (Cohen's d = 0.72). Observational and interview data revealed increased student engagement, collaboration, and real?world application of physics principles. Regression analysis indicated that initial academic performance and in-class engagement were significant predictors of post-test success (R^2^ = 0.65, *p* < 0.001), with VIF values below 2 confirming minimal multicollinearity.

**Conclusions:**

Findings suggest that PBL substantially enhances both cognitive and practical skills, fostering critical thinking and teamwork in university physics education. Practical implementation challenges—such as increased preparation time and the need for specialized facilitation—remain. Future research should explore the long-term impact of PBL in varied educational settings and its adaptability across disciplines.

## 1 Introduction

Project-Based Learning (PBL) originated in the 1970s within Danish engineering education and has evolved into a widely adopted pedagogical model in higher education. PBL emphasizes a learner-centered approach in which students engage in collaborative projects to tackle complex, real-world problems, thereby fostering deep engagement, and critical thinking (Blumenfeld et al., 1991; Helle et al., 2006). In recent years, its integration into university-level physics education has gained momentum as it facilitates the development of both conceptual understanding and hands-on, practical skills essential for the field (Bell, 2010; Krajcik and Shin, 2014; AlAli et al., 2024; English and King, 2017; Sullivan and Bers, 2019; Al-Kamzari and Alias, 2025).

The “General University Physics Curriculum Standards” (2018) issued by the Ministry of Education of the People's Republic of China, emphasizes the cultivation of critical thinking, problem-solving abilities, and innovation (Ministry of Education of the People's Republic of China, 2018). This paradigm shift—from traditional, teacher-centric pedagogies to more interactive, student-centered methods—is vital for meeting these educational objectives. PBL provides a robust framework for this transition by promoting continuous, project-based activities that immerse students in complex physics concepts while enabling the practical application of theoretical knowledge (Prince and Felder, 2006).

Within the realm of physics education, PBL fosters active student participation by engaging learners in processes such as formulating questions, gathering and analyzing data, and collaborating with peers. In such environments, educators transition from traditional lecturers to facilitators, guiding students through exploratory learning to acquire both advanced knowledge and practical skills (Helle et al., 2006). Empirical studies have consistently demonstrated that PBL not only enhances student engagement but also improves the development of practical skills, which has contributed to its growing adoption in physics courses (Blumenfeld et al., 1991). Moreover, the incorporation of digital tools and interdisciplinary strategies has further expanded the impact of PBL on student outcomes (Sari and Wilujeng, 2021; Sager et al., 2025; Jarrah et al., 2025).

### 1.1 Theoretical framework and hypotheses

PBL is grounded in constructivist learning theory, which asserts that learners actively construct knowledge through engagement and social interaction (Helle et al., 2006). Vygotsky's social constructivism further emphasizes the importance of collaborative learning (Krajcik and Shin, 2014; Spiteri et al., 2022), advocating that educators act as facilitators who support student inquiry rather than merely transmitting information (Krajcik and Shin, 2014). These theoretical perspectives underpin the effectiveness of PBL by suggesting that students are more likely to internalize and apply knowledge when engaged in meaningful, real-world tasks.

Based on these theoretical underpinnings, we propose the following hypotheses:

**1. Conceptual understanding:** PBL will enhance students' conceptual understanding of physics by engaging them in problem-solving activities that promote deeper comprehension of complex concepts.

**2. Problem-solving and practical skills:** PBL will improve students' problem-solving abilities and practical skills by enabling them to apply theoretical knowledge to real-world challenges.

**3. Engagement and motivation:** PBL will increase student engagement and motivation in learning physics, as its collaborative, inquiry-driven nature fosters sustained participation.

**4. Critical thinking and teamwork:** PBL will promote the development of critical thinking and teamwork skills, thereby enhancing students' ability to work effectively in collaborative settings.

A substantial body of research has demonstrated that PBL positively influences student motivation, engagement, and academic achievement in higher education (Bani Issa and Khataibeh, 2021; Preininger, 2017). Specifically, in the context of university physics education, PBL has been shown to improve conceptual understanding, problem-solving skills, and practical knowledge (Prince and Felder, 2006; Blumenfeld et al., 1991; Capraro et al., 2013; Vallera and Bodzin, 2019). Furthermore, by fostering critical thinking and collaborative competencies, PBL equips students with essential skills for success in scientific and interdisciplinary fields.

## 2 Methodology and data collection

This study adopted a robust mixed-methods approach to explore the impact of Project-Based Learning (PBL) on university students' comprehension of physics concepts, problem-solving abilities, practical skills, engagement, and motivation. The following sections detail the research design, participant selection and assignment, PBL implementation, data collection procedures, and data analysis techniques.

### 2.1 Research design

A quasi-experimental design was employed to collect both quantitative and qualitative data, offering a comprehensive view of PBL's effects. The quantitative component involved administering standardized pre- and post-tests to assess students' conceptual understanding and problem-solving proficiency. Simultaneously, qualitative data were obtained through structured classroom observations, semi-structured student interviews, and detailed analysis of project artifacts. These multiple data sources enabled a nuanced evaluation of student engagement, motivation, and practical skill development.

### 2.2 Participants and group assignment

The study involved 150 undergraduate physics students enrolled in introductory courses during the 2023–2024 academic year at three universities in Harbin, China. To ensure comparability, participants were first matched on key demographic variables and prior academic performance—including baseline physics knowledge measured by pre-test scores. Subsequently, a stratified randomization process was used to assign participants evenly into an experimental group (*n* = 75), which participated in the PBL intervention, and a control group (*n* = 75), which received traditional lecture-based instruction. This stratification ensured balance across variables such as gender, academic background, and baseline physics proficiency.

### 2.3 PBL implementation

Within the PBL framework, students engaged in a series of well-structured, contextually relevant projects designed to foster deep understanding of core physics concepts and enhance practical problem-solving skills. Project design was aligned with specific learning objectives outlined in the physics curriculum. Instructors provided clear guidelines and detailed rubrics while granting students a degree of autonomy to select project topics and propose modifications based on their interests. This approach balanced structured learning with creative student input. Throughout the project cycle, instructors acted as facilitators, offering ongoing feedback, ensuring access to necessary resources, and controlling for potential confounding factors such as varying levels of prior physics knowledge.

### 2.4 Types of projects

Projects spanned several fundamental physics topics, including:

#### 2.4.1 Wave interference and diffraction

Students investigated wave interference using a laser and a double-slit apparatus. They formulated hypotheses, conducted experiments, recorded observations, and analyzed results to calculate the laser's wavelength.

#### 2.4.2 Electrical circuit design

In this project, students designed and constructed an electrical circuit to control lighting based on ambient light levels. They researched sensor types, created circuit schematics, built the circuit, and tested its functionality. The iterative process encouraged troubleshooting and refinement.

#### 2.4.3 Thermodynamics and heat transfer

Students explored the energy efficiency of various insulation materials by designing experiments to measure heat transfer rates. Through data analysis, they determined the most effective insulation material and discussed its real-world applications, such as building design.

### 2.5 Assessment of student performance

Student performance was evaluated using a combination of quantitative and qualitative measures:

#### 2.5.1 Conceptual understanding

Standardized pre- and post-tests assessed the grasp of core physics concepts.

#### 2.5.2 Practical application

The quality and accuracy of project outputs (e.g., laboratory reports, presentations, and prototype designs) were analyzed to determine the ability to apply theoretical knowledge to practical problems.

#### 2.5.3 Collaboration and engagement

Classroom observations, combined with peer and instructor evaluations, provided insights into teamwork, communication, and overall engagement. Interaction frequencies and the quality of group discussions served as key indicators.

#### 2.5.4 Critical thinking and problem-solving

The depth of analytical reasoning and the ability to propose innovative solutions were systematically evaluated.

Instructors used detailed rubrics aligned with these criteria to ensure consistent and fair evaluation through both formative and summative assessment methods.

### 2.6 Data collection

Quantitative data:

#### 2.6.1 Pre-tests and post-tests

Standardized tests were administered in both the experimental and control groups before and after the intervention to measure students' conceptual understanding and problem-solving capabilities.

#### 2.6.2 Classroom observations

Structured observations were conducted in both PBL and traditional settings to document student interaction, participation levels, and teaching practices. An observation checklist was employed to ensure uniformity and reliability.

#### 2.6.3 Project work analysis

Students' project outputs, including reports, presentations, and other artifacts, were collected and evaluated to assess the application of physics concepts and practical skill development.

#### 2.6.4 Qualitative data

##### 2.6.4.1 Student interviews

Semi-structured interviews were conducted with selected students from the experimental group to gain insights into their PBL experiences, particularly regarding engagement, motivation, and skill development. Interviews were audio-recorded, transcribed verbatim, and later analyzed.

##### 2.6.4.2 Content analysis

The collected project artifacts underwent content analysis to evaluate creativity, understanding, and problem-solving strategies.

### 2.7 Data analysis

#### 2.7.1 Quantitative data analysis

##### 2.7.1.1 Descriptive statistics

Means, standard deviations, and percentage scores were calculated for the pre- and post-tests to summarize performance trends.

##### 2.7.1.2 Inferential statistics

Paired *t*-tests were used to assess score differences within each group, while independent *t*-tests compared performance between the experimental and control groups. A significance level of 0.05 was applied across all tests.

#### 2.7.2 Multiple regression analysis

To identify predictors of post-test scores, multiple regression analysis was performed. The model's R^2^ value (e.g., R^2^ = 0.65) was reported, and diagnostic tests for normality (e.g., Shapiro-Wilk test) and multicollinearity (e.g., Variance Inflation Factor, VIF <2 for all predictors) were conducted. Baseline physics knowledge was included as a control variable to account for its potential confounding effect.

#### 2.7.3 Qualitative data analysis

##### 2.7.3.1 Thematic analysis

Interview transcripts and observation notes underwent thematic analysis to uncover recurrent themes and patterns related to engagement, motivation, and skill development. Two independent researchers coded the data to ensure reliability.

##### 2.7.3.2 Content analysis

The project artifacts were examined using content analysis, focusing on the demonstration of physics concept application, creativity, and problem-solving strategies.

## 3 Results

### 3.1 Descriptive and inferential statistics on the impact of project-based learning (PBL) on students' conceptual understanding and problem-solving abilities

The post-test results were analyzed to assess the effectiveness of PBL in enhancing students' conceptual understanding and problem-solving skills. As shown in Table 1, both groups exhibited significant improvement in post-test scores; however, the experimental group that participated in PBL demonstrated a substantially greater increase compared to the control group. Specifically, the experimental group achieved an average increase of 19.9 points (SD = 9.2) compared to 6.6 points (SD = 8.3) in the control group. This difference was statistically significant (t = 12.35, *p* < 0.001), with a very large effect size (Cohen's d = 1.85) for the experimental group, in contrast to a moderate effect (Cohen's d = 0.72) observed in the control group. The large effect size indicates that the PBL intervention had a meaningful and practical impact on student learning outcomes.

**Table 1:** Descriptive and inferential statistics of the impact of PBL on students' conceptual understanding and problem-solving abilities.

**Group**	**Pre-test mean (SD)**	**Post-test mean (SD)**	**Mean difference (SD)**	**t-value**	***p*-value**	**Effect size (Cohen's d)**
Experimental	55.3 (10.4)	75.2 (12.3)	19.9 (9.2)	12.35	<0.001	1.85
Control	56.1 (9.8)	62.7 (10.5)	6.6 (8.3)	4.56	<0.001	0.72

### 3.2 Classroom observations

Classroom observations revealed marked differences in student engagement between the two groups. In PBL classrooms, students were observed engaging in higher levels of interaction and collaboration—frequently posing questions, engaging in discussions, and actively participating in group projects. In contrast, students in traditional lecture-based settings were more passive, with significantly fewer interactive behaviors. On average, the experimental group recorded 15.8 interactions per 30-min session compared to 5.3 interactions in the control group, as detailed in Table 2. These findings support the assertion that PBL environments stimulate greater student engagement.

**Table 2:** Classroom interaction frequency (interactions per 30-min session).

**Group**	**Mean interactions (SD)**
Experimental	15.8 (3.1)
Control	5.3 (1.8)

### 3.3 Student interviews

Thematic analysis of semi-structured interviews with students from the experimental group revealed several key themes:

#### 3.3.1 Increased engagement

Students reported feeling more motivated and actively involved in the learning process due to the hands-on, real-world problem-solving nature of PBL.

#### 3.3.2 Enhanced understanding

Many students noted that applying theoretical knowledge to practical tasks during projects significantly deepened their understanding of physics concepts.

#### 3.3.3 Improved collaboration skills

Students emphasized that PBL fostered improved teamwork and communication skills, which they attributed to the collaborative project-based approach.

Inter-rater reliability for the coding process was high, with a Cohen's kappa of 0.82, ensuring the credibility of the thematic analysis.

### 3.4 Project work analysis

Content analysis of students' project outputs provided further evidence of improved practical skills and conceptual application. The projects, which were evaluated using detailed rubrics, demonstrated not only creativity and innovative problem-solving strategies but also a strong integration of multiple physics concepts. The following case study highlights illustrate the outcomes:

#### 3.4.1 Case study 1: wave interference and diffraction

Students explored wave interference using a laser and double-slit apparatus. They formulated hypotheses, conducted experiments, and calculated the laser wavelength based on their observations, demonstrating a solid understanding of wave phenomena.

#### 3.4.2 Case study 2: electrical circuit design

In this project, students designed and constructed electrical circuits to control lighting. The iterative design process, which involved troubleshooting and refinement, enhanced their grasp of electrical concepts.

#### 3.4.3 Case study 3: thermodynamics and energy efficiency

Students examined the energy efficiency of different insulation materials by designing experiments to measure heat transfer rates. Their analysis not only identified the most effective material but also provided insights into practical applications in building insulation.

### 3.5 Multiple regression analysis

Multiple regression analysis was conducted to examine the relationship between demographic factors and post-test scores while controlling for potential confounding variables such as baseline physics knowledge. The model yielded a statistically significant overall effect with an R^2^ value of 0.65, indicating that 65% of the variance in post-test scores could be explained by the predictors. Diagnostic tests confirmed that the assumptions of normality (via Shapiro-Wilk test) and multicollinearity (all predictors exhibited VIF <2) were met. The analysis indicated that both initial academic performance and student engagement significantly predicted post-test scores. Moreover, the PBL intervention itself was a significant predictor, suggesting that students with higher initial engagement levels benefited more from the intervention (Table 3).

**Table 3:** Results of multiple regression analysis.

**Variable**	**Regression coefficient (B)**	**Standard error (SE)**	**t-value**	***p*-value**	**Beta**
Initial academic performance	0.35	0.08	4.38	<0.001	0.42
Student engagement	0.29	0.07	3.98	<0.001	0.38
PBL intervention	1.12	0.15	7.47	<0.001	0.62

### 3.6 Multivariate analysis of variance

A MANOVA was conducted to assess the overall effect of PBL on multiple dependent variables, including conceptual understanding, problem-solving abilities, and practical skills. The results confirmed that PBL had a significant positive impact across these learning outcomes, with moderate effect sizes observed. Table 4 provides a summary of the MANOVA findings, underscoring the broad benefits of the PBL approach.

**Table 4:** Multivariate analysis of variance (MANOVA) results.

**Dependent variable**	**Wilks' lambda**	**F-value**	***p*-value**	**Effect Size (η^2^)**
Conceptual understanding	0.85	5.12	<0.01	0.15
Problem-solving abilities	0.80	6.89	<0.001	0.20
Practical skills	0.88	4.50	<0.05	0.12

## 4 Discussion

The findings from this study offer compelling evidence supporting the positive impact of Project-Based Learning (PBL) on university students' comprehension of physics concepts, problem-solving abilities, practical skills, engagement, and motivation. This section situates these results within the broader literature, discusses educational implications, and addresses the challenges and limitations associated with the implementation of PBL, while also offering concrete recommendations for future practice and research.

### 4.1 Impact on conceptual understanding and problem-solving abilities

The significant improvement in post-test scores observed among students in the experimental group highlights PBL's effectiveness in promoting a deeper understanding of complex physics concepts and enhancing problem-solving skills. The large effect size (Cohen's d = 1.85) demonstrates that PBL had a much more substantial impact on learning outcomes compared to traditional lecture-based approaches (Cohen's d = 0.72). These findings align with previous studies that emphasize the role of PBL in promoting comprehensive learning outcomes through hands-on, real-world problem-solving tasks (Blumenfeld et al., 1991; Prince and Felder, 2006; Nurhayati and Suherman, 2021; Mota et al., 2025). By actively engaging students in the application of theoretical knowledge, PBL bridges the gap between abstract physics concepts and practical applications, fostering deeper cognitive engagement (Chen et al., 2025; D'Elia et al., 2025; Gümüş and Bozkurt Altan, 2025).

### 4.2 Enhancement of practical skills and engagement

Quantitative and qualitative findings converge to demonstrate that PBL not only increases student engagement—as evidenced by the significantly higher interaction frequency in PBL classrooms—but also promotes practical skill development. Students in the PBL group were better able to apply physics concepts in meaningful, real-world contexts. Furthermore, qualitative interviews revealed that the collaborative and hands-on nature of PBL nurtured essential soft skills, including teamwork, communication, leadership, and adaptability (Al-Kamzari and Alias, 2025). These transferable competencies are critical not only for success in physics but also across a variety of professional fields, thereby enhancing the overall value of PBL in higher education (Al-Kamzari and Alias, 2025).

### 4.3 Development of collaborative and critical thinking skills

One of the primary advantages of PBL is its capacity to cultivate students' collaborative and critical thinking skills. Participants reported improved abilities to work effectively in teams, share responsibilities, and jointly solve complex problems. Such skills are invaluable in scientific and interdisciplinary contexts, where teamwork and innovative problem-solving are paramount (Zhang and Ma, 2023). The significant gains observed in both cognitive and collaborative domains underscore that PBL equips students with the transferable skills essential for long-term professional development (Zhang et al., 2023).

### 4.4 Challenges and considerations for implementation

Despite its many benefits, implementing PBL presents several challenges (Lim et al., 2020; McGrath and Harris, 2020). One of the primary difficulties is the extensive preparation time required to design and facilitate effective PBL activities. Instructors must transition from traditional lecturers to facilitators, necessitating comprehensive professional development and pedagogical adaptation. For instance, institutions should consider providing dedicated training workshops and allocating sufficient time and resources for teachers to develop and refine PBL curricula (Bani Issa and Khataibeh, 2021). Additionally, students who are accustomed to passive learning may initially resist the active, self-directed nature of PBL. To mitigate this, a gradual introduction of PBL—supported by clear guidelines, mentorship, and institutional backing—is recommended (Zhang et al., 2023).

### 4.5 Alignment with curriculum standards and assessment practices

Maximizing the effectiveness of PBL requires careful alignment with established curriculum standards and robust assessment frameworks. This study demonstrates that PBL projects designed with clear rubrics and well-defined learning objectives not only foster deep conceptual understanding and practical skill development but also ensure that students meet national educational standards (Ministry of Education of the People's Republic of China, 2018; Zhang et al., 2023). The use of detailed rubrics enables a more meaningful and objective assessment of student progress across cognitive and practical domains (Nurhayati and Suherman, 2021; Mota et al., 2025).

### 4.6 Future research directions

While this study provides robust evidence for the immediate benefits of PBL, future research should investigate its long-term effects. Longitudinal studies could explore how PBL influences students' academic performance and career trajectories over time (D'Elia et al., 2025; Gümüş and Bozkurt Altan, 2025). Furthermore, it is essential to examine the effectiveness of PBL across diverse educational settings and student populations, particularly those from underrepresented or disadvantaged backgrounds (Zhang et al., 2023). Tailoring PBL to meet the needs of diverse learners is critical for ensuring its broader applicability and impact. Additionally, the integration of digital tools and technology within PBL frameworks presents a promising avenue for enhancing student engagement and facilitating collaborative learning, especially in remote or hybrid learning environments (Park and Kim, 2019; Yulianti and Herman, 2023).

### 4.7 Course design principles

The successful implementation of PBL in university physics education depends on adhering to several key design principles:

#### 4.7.1 Realism and relevance

PBL projects should be grounded in real-world contexts that enable students to connect theoretical knowledge to practical applications. In this study, projects related to wave interference and thermodynamics allowed students to explore physics phenomena with tangible real-world implications (Husin et al., 2025; Lenz et al., 2015).

#### 4.7.2 Alignment with curriculum standards

PBL activities must align with educational standards and clearly defined learning objectives to ensure that students acquire the necessary skills and knowledge (Ministry of Education of the People's Republic of China, 2018).

#### 4.7.3 Operability and focus

Given time constraints, PBL activities should be manageable within class hours and available resources. Well-structured projects can maximize engagement while minimizing excessive out-of-class workload.

#### 4.7.4 Supportive environment

A supportive classroom environment is crucial. Educators should act as facilitators, providing ongoing guidance and feedback. In this study, instructors played a pivotal role in helping students navigate complex problems and effectively apply physics concepts (Rehman et al., 2025; Yousef and Ayyoub, 2024; Makamure, 2025).

By adhering to these design principles, educators can optimize the benefits of PBL, creating a more engaging, interactive learning environment that supports both academic achievement and the development of practical, transferable skills.

## 5 Conclusion

This study provides compelling evidence that Project-Based Learning (PBL) significantly enhances university students' conceptual understanding, problem-solving abilities, practical skills, engagement, and motivation in the context of physics education. The findings demonstrate that PBL not only deepens comprehension of complex physics concepts but also fosters essential soft skills, such as teamwork, communication, and leadership—skills that are crucial in both academic and professional settings. However, to fully realize its potential, challenges related to curriculum alignment and teacher support must be addressed. Structured PBL activities that conform to national educational standards and include comprehensive teacher training are key to maximizing its effectiveness. Future research should focus on the long-term impacts of PBL and explore its cross-disciplinary applications to further validate its efficacy as a powerful educational tool.

## Data Availability

The datasets generated and analyzed during the current study are available from the corresponding author on reasonable request.
